# PREVALENCE AND CORRELATES OF SYMPTOMS OF ANXIETY AND DEPRESSION AT THE VERY END OF LIFE

**DOI:** 10.1093/geroni/igz038.2475

**Published:** 2019-11-08

**Authors:** Elissa K Kozlov, Veerawat Phongtankuel, Cary Reid

**Affiliations:** 1 Rutgers University, New Brunswick, New Jersey, United States; 2 Weill Cornell Medicine, New York, New York, United States

## Abstract

Rates of psychological symptoms for patients with serious illness is high, but there has been limited research investigating psychological symptoms at the very end of life. The aim of this study was to better understand the prevalence, intensity and correlates of psychological distress at the very end of life. This cross-sectional study utilized caregiver proxy interviews. Caregivers were contacted after their loved one recently died after being on home hospice and invited to participate in a brief interview with a trained research assistant. Patient, caregiver and hospice utilization data were also abstracted from electronic medical records. N = 351 caregivers were included in the study. According to caregivers, 46.4% of patients had moderate to severe anxiety, as assessed with a score of ≥4 on the Edmonton Symptom Assessment Scale (ESAS) and 43% had moderate to severe symptoms of depression in the last week of life. Symptoms of anxiety and depression were significantly associated with caregiver burden scores and inversely associated with the age of the patient. Psychological symptom management at the very end of life is essential to providing comprehensive hospice care. Our study revealed that nearly half of patients die with moderate to severe symptoms of anxiety and/or depression. Future research is needed to improve psychological symptom management at the very end of life in order to improve the quality of life for both patients and their families.

